# Transactions of the American Dermatological Association at the Twenty-fourth Annual Meeting, Held in Washington, D. C., May 1, 2 and 3, 1900, in Connection with the Fifth Triennial Session of the Congress of American Physicians and Surgeons

**Published:** 1901-12

**Authors:** 


TRANSACTIONS of the American Dermatological Association at the Twenty-fourth Annual Meeting, held in Washington, D. C., May 1, 2 and 3, 1900, in connection with the Fifth Triennial Session of the Congress of American Physicians and Surgeons. Official Report of the Proceedings, by FRANK HUGH MONTGOMERY, Secretary. Chicago: P. F. Pettibone. 1901.

The American Dermatological Association may be proud of the work done at the session which was held in connection with the fifth Triennial Congress of American Physicians and Surgeons. The proceedings form a welcome addition to dermatological literature and plainly show the progress made by American physicians in this special branch of medicine.

The work consists of 232 pages and contains numerous illustrations, including reproductions of histological preparations. It is, of course, impossible to review in detail the papers and subsequent discussions. In the annual address made by the president, Dr. Stelwagon, he formulated a plea for greater attention upon the part of the dermatologist to bacteriology, which seems to be practically useless on account of the easy contamination. It is well known that, in a bacteriological sense, we can find almost anything we look for in the skin. Dr. Stelwagon, however, made a statement which should be recognised as important by every university,—that dermatology should have a far more prominent place in the medical curriculum, and that each large hospital should devote a special ward to the treatment of skin diseases. Another paper of interest was by Dr. Corlett, upon The frequency of parasitic diseases of the skin, and measures advisable for limiting their spread, in which he recommends, as a preventative to ringworm, that there should be medical inspectors for schools who should inspect the children regularly and isolate the child infected to a place where its education may be continued without loss of time. He also considers that barber-shops should be under the supervision of the Board of Health and that it should be illegal for a barber to use the same brush, sponge, towel or instrument on different persons without first well caring for them.

The general character of the papers was elevated and the numerous drawings and photographs presented for inspection were unusually excellent. As a whole, the session reported in the volume was one of remarkable interest and advantage.

